# Phenome-wide association analysis of LDL-cholesterol lowering genetic variants in *PCSK9*

**DOI:** 10.1186/s12872-019-1187-z

**Published:** 2019-10-29

**Authors:** Amand F. Schmidt, Michael V. Holmes, David Preiss, Daniel I. Swerdlow, Spiros Denaxas, Ghazaleh Fatemifar, Rupert Faraway, Chris Finan, Dennis Valentine, Zammy Fairhurst-Hunter, Fernando Pires Hartwig, Bernardo Lessa Horta, Elina Hypponen, Christine Power, Max Moldovan, Erik van Iperen, Kees Hovingh, Ilja Demuth, Kristina Norman, Elisabeth Steinhagen-Thiessen, Juri Demuth, Lars Bertram, Christina M. Lill, Stefan Coassin, Johann Willeit, Stefan Kiechl, Karin Willeit, Dan Mason, John Wright, Richard Morris, Goya Wanamethee, Peter Whincup, Yoav Ben-Shlomo, Stela McLachlan, Jackie F. Price, Mika Kivimaki, Catherine Welch, Adelaida Sanchez-Galvez, Pedro Marques-Vidal, Andrew Nicolaides, Andrie G. Panayiotou, N. Charlotte Onland-Moret, Yvonne T. van der Schouw, Giuseppe Matullo, Giovanni Fiorito, Simonetta Guarrera, Carlotta Sacerdote, Nicholas J. Wareham, Claudia Langenberg, Robert A. Scott, Jian’an Luan, Martin Bobak, Sofia Malyutina, Andrzej Pająk, Ruzena Kubinova, Abdonas Tamosiunas, Hynek Pikhart, Niels Grarup, Oluf Pedersen, Torben Hansen, Allan Linneberg, Tine Jess, Jackie Cooper, Steve E. Humphries, Murray Brilliant, Terrie Kitchner, Hakon Hakonarson, David S. Carrell, Catherine A. McCarty, Kirchner H. Lester, Eric B. Larson, David R. Crosslin, Mariza de Andrade, Dan M. Roden, Joshua C. Denny, Cara Carty, Stephen Hancock, John Attia, Elizabeth Holliday, Rodney Scott, Peter Schofield, Martin O’Donnell, Salim Yusuf, Michael Chong, Guillaume Pare, Pim van der Harst, M. Abdullah Said, Ruben N. Eppinga, Niek Verweij, Harold Snieder, Lifelines Cohort authors, Tim Christen, D. O. Mook-Kanamori, the ICBP Consortium, Stefan Gustafsson, Lars Lind, Erik Ingelsson, Raha Pazoki, Oscar Franco, Albert Hofman, Andre Uitterlinden, Abbas Dehghan, Alexander Teumer, Sebastian Baumeister, Marcus Dörr, Markus M. Lerch, Uwe Völker, Henry Völzke, Joey Ward, Jill P. Pell, Tom Meade, Ingrid E. Christophersen, Anke H. Maitland-van der Zee, Ekaterina V. Baranova, Robin Young, Ian Ford, Archie Campbell, Sandosh Padmanabhan, Michiel L. Bots, Diederick E. Grobbee, Philippe Froguel, Dorothée Thuillier, Ronan Roussel, Amélie Bonnefond, Bertrand Cariou, Melissa Smart, Yanchun Bao, Meena Kumari, Anubha Mahajan, Jemma C. Hopewell, Sudha Seshadri, the METASTROKE Consortium of the ISGC, Caroline Dale, Rui Providencia E. Costa, Paul M. Ridker, Daniel I. Chasman, Alex P. Reiner, Marylyn D. Ritchie, Leslie A. Lange, Alex J. Cornish, Sara E. Dobbins, Kari Hemminki, Ben Kinnersley, Marc Sanson, Karim Labreche, Matthias Simon, Melissa Bondy, Philip Law, Helen Speedy, James Allan, Ni Li, Molly Went, Niels Weinhold, Gareth Morgan, Pieter Sonneveld, Björn Nilsson, Hartmut Goldschmidt, Amit Sud, Andreas Engert, Markus Hansson, Harry Hemingway, Folkert W. Asselbergs, Riyaz S. Patel, Brendan J. Keating, Naveed Sattar, Richard Houlston, Juan P. Casas, Aroon D. Hingorani

**Affiliations:** 1 Institute of Cardiovascular Science, University College London, 222 Euston Road, London, NW1 2DA UK; 2 Department of Cardiology, Division Heart & Lungs, University Medical Center Utrecht, Utrecht University, Utrecht, the Netherlands; 3 UCL’s BHF Research Accelerator Centre, London, UK; 4 Medical Research Council Population Health Research Unit, Clinical Trial Service Unit & Epidemiological Studies Unit (CTSU), Nuffield Department of Population Health, University of Oxford, Oxford, UK; 5 Department of Medicine, Imperial College London, London, UK; 6 Health Data Research UK, University College London, 222 Euston Road, London, NW1 2DA UK; 7 Institute of Health Informatics, University College London, 222 Euston Road, London, NW1 2DA UK; 8 The Alan Turing Institute, British Library, 96 Euston Rd, London, NW1 2DB UK; 9 University College London, Farr Institute of Health Informatics, London, UK; 10 Clinical Trial Service Unit & Epidemiological Studies Unit (CTSU), Nuffield Department of Population Health, University of Oxford, Richard Doll Building, Old Road Campus, Roosevelt Drive, Oxford, OX3 7LF UK; 11 Postgraduate Program in Epidemiology, Federal University of Pelotas, Pelotas, Brazil; 12 Centre for Population Health Research, Sansom Institute for Health Research, University of South Australia, Adelaide, Australia; 13 Population, Policy and Practice, UCL GOS Institute of Child Health, London, UK; 14 South Australian Health and Medical Research Institute, Adelaide, Australia; 15 Durrer Center for Cardiovascular Research, Netherlands Heart Institute, Utrecht, The Netherlands; 16 Department of Clinical Epidemiology, Biostatistics And Bioinformatics, Academic Medical Center Amsterdam, Amsterdam, the Netherlands; 17 Department of vascular medicine, Academic Medical Center Amsterdam, Amsterdam, the Netherlands; 18 Charité – Universitätsmedizin Berlin, corporate member of Freie Universität Berlin, Humboldt-Universität zu Berlin, and Berlin Institute of Health, Lipid Clinic at the Interdisciplinary Metabolism Center, Berlin, Germany; 19 Charité - Universitätsmedizin Berlin, BCRT - Berlin Institute of Health Center for Regenerative Therapies, Berlin, Germany; 20 Institute of Nutritional Science, University of Potsdam, 14558 Nuthetal, Germany; 21 Geriatrics Research Group, Charité - Universitätsmedizin Berlin, 13347 Berlin, Germany; 22 Department of Nutrition and Gerontology, German Institute of Human Nutrition Potsdam-Rehbruecke, 14558 Nuthetal, Germany; 23 E.CA Economics GmbH, Berlin, Germany; 24 Lübeck Interdisciplinary Platform for Genome Analytics (LIGA), Institutes of Neurogenetics & Cardiogenetics, University of Lübeck, Lübeck, Germany; 25 Center for Lifespan Changes in Brain and Cognition (LCBC), Dept. Psychology, University of Oslo, Oslo, Norway; 26 Genetic and Molecular Epidemiology Group, Lübeck Interdisciplinary Platform for Genome Analytics (LIGA), Institutes of Neurogenetics & Cardiogenetics, University of Lübeck, Lübeck, Germany; 27 Institute of Human Genetics, Lübeck, Germany; 28 Ageing Epidemiology Research Unit, School of Public Health, Imperial College, London, UK; 29 Institute of Genetic Epidemiology, Department of Genetics and Pharmacology, Medical University of Innsbruck, 6020 Innsbruck, Austria; 30 Department of Neurology, Medical University Innsbruck, Innsbruck, Austria; 31 Department of Neurology, Inselspital, University Hospital Bern, University of Bern, Bern, Switzerland; 32 Bradford Institute for Health Research, Bradford Royal Infirmary, Bradford, UK; 33 Department Primary Care & Population Health, University College London, London, UK; 34 Population Health Research Institute, St George’s, University of London, London, UK; 35 Population Health Sciences, University of Bristol, Bristol, UK; 36 Centre for Population Health Sciences, The Usher Institute, University of Edinburgh, Edinburgh, UK; 37 Department of Epidemiology and Public Health, UCL Institute of Epidemiology and Health Care, University College London, London, UK; 38 Department of Medicine, Internal Medicine, Lausanne University Hospital, Lausanne, Switzerland; 39 Department of Vascular Surgery, Imperial College, London, United Kingdom; 40 Department of Surgery, Nicosia Medical School, University of Nicosia, Nicosia, Cyprus; 41 Cyprus International Institute for Environmental and Public Health, Cyprus University of Technology, Limassol, Cyprus; 42 Julius Center for Health Sciences and Primary Care, University Medical Center Utrecht, Utrecht University, Utrecht, the Netherlands; 43 Italian Institute for Genomic Medicine (IIGM), Turin, Italy; 44 Department of Medical Sciences, University of Turin, Turin, Italy; 45 Unit of Cancer Epidemiology, Città della Salute e della Scienza University-Hospital and Center for Cancer Prevention (CPO), Turin, Italy; 46 MRC Epidemiology Unit, Institute of Metabolic Science, University of Cambridge School of Clinical Medicine, Cambridge Biomedical Campus, Addenbrooke’s Hospital, Cambridge, UK; 47 Novosibirsk State Medical University, Novosibirsk, Russian Federation; 48 Institute of Internal and Preventive Medicine, Siberian Branch of the Russian Academy of Medical Sciences, Novosibirsk, Russian Federation; 49 Department of Epidemiology and Population Studies, Faculty of Health Sciences, Jagiellonian University Medical College, Kraków, Poland; 50 National Institute of Public Health, Prague, Czech Republic; 51 Lithuanian University of Health Sciences, Kaunas, Lithuania; 52 Novo Nordisk Foundation Center for Basic Metabolic Research, Faculty of Health and Medical Sciences, University of Copenhagen, Copenhagen, Denmark; 53 Department of Clinical Medicine, Faculty of Health and Medical Sciences, University of Copenhagen, Copenhagen, Denmark; 54 Center for Clinical Research and Prevention, Bispebjerg and Frederiksberg Hospital, Copenhagen, The Capital Region of Denmark Denmark; 55 Centre for Cardiovascular Genetics, Department of Medicine, University College London, London, UK; 56 Center for Human Genetics, Marshfield Clinic Research Institute, Marshfield, USA; 57 Children’s Hospital of Philadelphia, Philadelphia, USA; 58 University of Minnesota, Minneapolis, USA; 59 Geisinger, Danville, USA; 60 Kaiser Permanente Washington Health Research Institute, Seattle, WA USA; 61 Department of Biomedical Informatics and Medical Education University of Washington Seattle, Seattle, WA USA; 62 Mayo Clinic, Rochester, USA; 63 Department of Medicine, Department of Pharmacology, Department of Biomedical Informatics, Vanderbilt University School of Medicine, Nashville, TN USA; 64 Vanderbilt University, Nashville, USA; 65 WHI, Seattle, USA; 66 University of Newcastle, Newcastle, NSW Australia; 67 Public Health Program, Hunter Medical Research Institute, Newcastle, NSW Australia; 68 Hunter New England Local Health District, Newcastle, NSW Australia; 69 Population Health Research Institute, Hamilton, Ontario Canada; 70 Department of Genetics, University of Groningen, University Medical Center Groningen, Groningen, The Netherlands; 71 Department of Cardiology, University of Groningen, University Medical Center Groningen, Groningen, The Netherlands; 72 Department of Epidemiology, University of Groningen, University Medical Center Groningen, Groningen, Netherlands; 73 Department of Clinical Epidemiology, Leiden University Medical Center, Leiden, The Netherlands; 74 Department of Medical Sciences, Molecular Epidemiology, Uppsala University, Uppsala, Sweden; 75 Department of Medicine, Division of Cardiovascular Medicine, Stanford University School of Medicine, Stanford, CA 94305 USA; 76 Department of Medical Sciences, Molecular Epidemiology and Science for Life Laboratory, Uppsala University, Uppsala, Sweden; 77 Department of Epidemiology, Erasmus University Medical Center, Rotterdam, the Netherlands; 78 Department of Epidemiology and Biostatistics, Imperial College London, London, UK; 79 Institute for Community Medicine, University Medicine Greifswald, Greifswald, Germany; 80 DZHK (German Centre for Cardiovascular Research), partner site Greifswald, Greifswald, Germany; 81 Chair of Epidemiology, Ludwig-Maximilians-Universität München, UNIKA-T Augsburg, Augsburg, Germany; 82 Department of Internal Medicine B, University Medicine Greifswald, Greifswald, Germany; 83 Department of Internal Medicine A, University Medicine Greifswald, Greifswald, Germany; 84 Interfaculty Institute of Genetics and Functional Genomics, University Medicine Greifswald, Greifswald, Germany; 85 Institute of Health and Wellbeing, University of Glasgow, Glasgow, G12 8RZ Scotland, UK; 86 Department of Non-Communicable Disease Epidemiology, London School of Hygiene & Tropical Medicine, London, UK; 87 The Department of Medical Research, Bærum Hospital, Vestre Viken Hospital Trust, Gjettum, Norway; 88 Division of Pharmacoepidemiology and Clinical Pharmacology, Utrecht Institute of Pharmaceutical Sciences, Faculty of Science, Utrecht University, Utrecht, The Netherlands; 89 Department of Respiratory Medicine, Academic Medical Centre, University of Amsterdam, Amsterdam, the Netherlands; 90 Robertson Centre for Biostatistics, University of Glasgow, Glasgow, UK; 91 Institute of Genetics and Molecular Medicine, University of Edinburgh, Edinburgh, UK; 92 Institute of Cardiovascular and Medical Sciences, University of Glasgow, Glasgow, G12 8TA UK; 93 CNRS UMR 8199, European Genomic Institute for Diabetes (EGID), Institut Pasteur de Lille, University of Lille, 59000 Lille, France; 94 Department of Genomics of Common Disease, Imperial College London, W12 0NN London, United Kingdom; 95 INSERM, U-1138, Centre de Recherche des Cordeliers, Paris, France; 96 UFR de Médecine, Université Paris Diderot, Sorbonne Paris Cité, Paris, France; 97 Départment de Diabétologie, Endocrinologie et Nutrition, Assistance Publique Hôpitaux de Paris, Hôpital Bicha, Paris, France; 98 l’institut du Thorax, INSERM, CNRS, UNIV Nantes, CHU Nantes, Nantes, France; 99 Institute for Social and Economic Research, University of Essex, Essex, UK; 100 Wellcome Trust Centre for Human Genetics, University of Oxford, Oxford, England; 101 Boston University School of Medicine, Boston, MA USA; 102 Harvard Medical School Center for Cardiovascular Disease Prevention Brigham and Women’s Hospital, Boston, USA; 103 UWash, Seattle, USA; 104 Penn State, State College, USA; 105 University of Colorado Denver, Denver, USA; 106 Division of Genetics and Epidemiology, The Institute of Cancer Research, London, UK; 107 Div. Molecular Genetic Epidemiology German Cancer Research Center, Im Neuenheimer Feld 580, 69120 Heidelberg, Germany; 108 Deutsches Krebsforschungszentrum, Heidelberg, Germany; 109 The Institut du Cerveau et de la Moelle épinière – ICM, Paris, France; 110 Sorbonne Universités, UPMC Université Paris 06, UMR S 1127, F-75013 Paris, France; 111 Department of Neurosurgery, Bethel Clinic, Kantensiek 11, 33617 Bielefeld, Germany; 112 Division of Hematology-Oncology, Department of Pediatrics, Dan L. Duncan Cancer Center, Baylor College of Medicine, Houston, Texas 77030 USA; 113 Northern Institute for Cancer Research, Newcastle University, Newcastle upon Tyne, UK; 114 Myeloma Institute for Research and Therapy, University of Arkansas for Medical Sciences, Little Rock, USA; 115 Department of Hematology, Erasmus MC Cancer Institute, 3075 EA Rotterdam, the Netherlands; 116 Hematology and Transfusion Medicine, Department of Laboratory Medicine, BMC B13, SE-221 84 Lund, Sweden; 117 University Clinic Heidelberg, Internal Medicine V and National Center for Tumor Diseases (NCT), Heidelberg, Germany; 118 Department of Internal Medicine, University Hospital of Cologne, Cologne, Germany; 119 Hematology Clinic, Skåne University Hospital, Skåne, Sweden; 120 Wallenberg Center for Molecular Medicine, Lund University, Lund, Sweden; 121 The National Institute for Health Research University College London Hospitals Biomedical Research Centre, University College London, 222 Euston Road, London, NW1 2DA UK; 122 Health Data Research UK and Institute of Health Informatics, University College London, London, United Kingdom; 123 The Barts Heart Centre, St Bartholomew’s Hospital, London, UK; 124 UPenn, Philadelphia, USA; 125 Massachusetts Veterans Epidemiology and Research Information Center (MAVERIC) Veterans Affairs Boston Healthcare System, Boston, USA

**Keywords:** Genetic association studies, Mendelian randomisation, LDL-cholesterol, Phenome-wide association scan

## Abstract

**Background:**

We characterised the phenotypic consequence of genetic variation at the *PCSK9* locus and compared findings with recent trials of pharmacological inhibitors of PCSK9.

**Methods:**

Published and individual participant level data (300,000+ participants) were combined to construct a weighted *PCSK9* gene-centric score (GS). Seventeen randomized placebo controlled PCSK9 inhibitor trials were included, providing data on 79,578 participants. Results were scaled to a one mmol/L lower LDL-C concentration.

**Results:**

The *PCSK9* GS (comprising 4 SNPs) associations with plasma lipid and apolipoprotein levels were consistent in direction with treatment effects. The GS odds ratio (OR) for myocardial infarction (MI) was 0.53 (95% CI 0.42; 0.68), compared to a PCSK9 inhibitor effect of 0.90 (95% CI 0.86; 0.93). For ischemic stroke ORs were 0.84 (95% CI 0.57; 1.22) for the GS, compared to 0.85 (95% CI 0.78; 0.93) in the drug trials. ORs with type 2 diabetes mellitus (T2DM) were 1.29 (95% CI 1.11; 1.50) for the GS, as compared to 1.00 (95% CI 0.96; 1.04) for incident T2DM in PCSK9 inhibitor trials. No genetic associations were observed for cancer, heart failure, atrial fibrillation, chronic obstructive pulmonary disease, or Alzheimer’s disease – outcomes for which large-scale trial data were unavailable.

**Conclusions:**

Genetic variation at the *PCSK9* locus recapitulates the effects of therapeutic inhibition of PCSK9 on major blood lipid fractions and MI. While indicating an increased risk of T2DM, no other possible safety concerns were shown; although precision was moderate.

**Electronic supplementary material:**

The online version of this article (10.1186/s12872-019-1187-z) contains supplementary material, which is available to authorized users.

## Background

Statins and ezetimibe reduce the risk of major coronary events and ischemic stroke via lowering of low density lipoprotein-cholesterol (LDL-C) [1–3]. Loss-of-function mutations in *PCSK9* are associated with lower LDL-C and a reduced risk of coronary heart disease (CHD) [4, 5]. Antibodies (mAbs) inhibiting PCSK9, reduce LDL-C in patients with hypercholesterolaemia, and received market access in 2015. The FOURIER and ODYSSEY OUTCOMES trials tested the efficacy of PCSK9-inhibition versus placebo on the background of statin treatment and both found that PCSK9 inhibition led to a 15% relative risk reduction of major vascular events in patients with established CVD and recent acute coronary syndrome over a median follow up of 2.2 to 2.8 years [6, 7].

Evidence is limited on the effect of PCSK9 inhibition on clinical outcomes, and on safety outcomes that might only become apparent with prolonged use. Nor is evidence available on the efficacy and safety of PCSK9 inhibitors in subjects other than the high-risk patients studied in trials. Mendelian randomisation for target validation uses naturally-occurring variation in a gene encoding a drug target to identify mechanism-based consequences of pharmacological modification of the same target [8]. Such studies have previously proved useful in predicting success and failure in clinical trials and have assisted in delineating on-target from off-target actions of first-in-class drugs [9–13]. For example, previous studies showed that variants in *HMGCR,* encoding the target for statins, were associated with lower concentrations of LDL-C and lower risk of coronary heart disease [9] (CHD), while confirming the on-target nature of the effect of statins on higher body weight and higher risk of type 2 diabetes (T2DM) [9].

We characterised the phenotypic consequences of genetic variation at *PCSK9* in a large, general population sample focussing on therapeutically relevant biomarkers, cardiovascular disease (CVD), individual CVD components and non-CVD outcomes such as cancer, Alzheimer’s disease, and chronic obstructive pulmonary disease (COPD). Effect estimates from the genetic analysis were compared to those from intervention trials where the outcomes under evaluation overlapped.

## Methods

We summarise methods briefly here as they have been previously described in detail [14].

### Genetic variant selection

SNPs rs11583680 (minor allele frequency [MAF] = 0.14), rs11591147 (MAF = 0.01), rs2479409 (MAF = 0.36) and rs11206510 (MAF = 0.17) were selected as genetic instruments at the *PCSK9* locus based on the following criteria: (1) an LDL-C association as reported by the Global Lipids Genetics Consortium (GLGC) [15]; (2) low pairwise linkage disequilibrium (LD) (*r*^2^ ≤ 0.30) with other SNPs in the region (based on 1000 Genomes CEU data); and (3) the combined annotation dependent depletion (CADD) score [16] which assesses potential functionality (see Additional file 1: Table S1).

Previously, we explored the between-SNP correlations (see Additional file 2: Figure S1 of Schmidt et al. 2017 [14]), revealing an $r^2$ of 0.26 between rs11206510 and rs11583680, confirming all other SNPs were approximately independent (*r*^2^ ≤ 0.07). Subsequent adjustment for the residual LD (correlation) structure did not impact results (see Appendix Figure 90 of Schmidt et al. 2017 [14]).

### Individual participant-level and summary-level data

Participating studies (Additional file 1: Table S2) provided analyses of individual participant-level data (IPD) based on a common analysis script (available from AFS), submitting summary estimates to the UCL analysis centre. These data were supplemented with public domain data from relevant genetic consortia (Additional file 1: Table S3). Studies contributing summary estimates to genetic consortia were excluded from the IPD component of the analysis to avoid duplication.

Biomarker data were collected on the major routinely measured blood lipids (LDL-C, HDL-C, triglycerides [TG], total cholesterol [TC]); apolipoproteins A1 [ApoA1] and B [ApoB], and nominal lipoprotein (Lp)(a); systolic (SBP) and diastolic (DBP) blood pressure; inflammation markers C-reactive protein (CRP), interleukin-6 (IL-6), and fibrinogen; haemoglobin; glycated haemoglobin (HbA_1c_); liver enzymes gamma-glutamyltransferase (GGT), alanine aminotransferase (ALT), aspartate transaminase (AST), and alkaline phosphatase (ALP); serum creatinine, and cognitive function (standardized to mean 0, and standard deviation 1, see Additional file 1: Table S5).

We focussed on individual clinical endpoints, rather than composites, which have been assessed in outcome trials, as well as disease end-points commonly seen in patients likely to be eligible for PCSK9 inhibitor treatment. Ischemic CVD endpoints studied were myocardial infarction (MI), ischemic stroke, revascularization, and angina. The following non-ischemic CVD events were considered: haemorrhagic stroke, heart failure, and atrial fibrillation. Non-CVD outcome data was collected on common chronic diseases: COPD, any cancer (including those of the breast, prostate, colon and lung), Alzheimer’s disease, and T2DM. Study endpoints and biomarker were chosen based on a combination of 1) available sample size, 2) clinical relevance, and 3) evaluation in RCTs of PCSK9 inhibition, we did not a priori hypothesize on the likelihood of *PCSK9* being associated with any of the available phenotypes. Specific cancer sites evaluated here: chronic lymphocytic leukaemia, multiple myeloma, Hodgkin, meningioma, glioma, melanoma, colorectal cancer, prostate cancer, breast cancer, lung adenocarcinoma, and small-cell lung cancer.

Finally, aggregated trial data on the effect of monoclonal PCSK9 (13 alirocumab trials, and 4 evolocumab trials) inhibitors were compared to placebo for MI, revascularization, ischemic or haemorrhagic stroke, cancer, and T2DM abstracted from the Cochrane systematic review [6, 17], with the addition of the OUTCOMES alirocumab trial published afterwards [18]. We compared effects on biomarkers and clinical endpoints common to both the genetic analysis and trials.

### Statistical analyses

In all analyses, we assumed an additive allelic effect with genotypes coded as 0, 1 and 2, corresponding to the number of LDL-C lowering alleles; model comparison tests did not show signs of non-additivity [14]. Continuous biomarkers were analysed using linear regression and binary endpoints using logistic regression. Study-specific associations were pooled for each SNP using the inverse variance weighted method for fixed effect meta-analysis. Study-specific associations were excluded if the SNP was not in Hardy-Weinberg equilibrium (see Additional file 1: Table S4, based on a Holm-Bonferroni alpha criterion), with no variants failing this test. We estimated the effect at the *PCSK9* locus by combining all four SNPs in a gene centric score (GS) as the inverse variance weighted effect of the 4 variants, that were subsequently scaled by the inverse variance weighted effect on LDL-C.

Trial data were assembled as per Schmidt et al. 2017 [6]. Briefly, systematic searches were performed using the Cochrane Central Register of Controlled Trials (CENTRAL), MEDLINE, Embase, Web of Science registries, Clinicaltrials.gov and the International Clinical Trials Registry Platform databases. Data from placebo controlled trials were extracted and combined using the inverse variance weighted method for continuous data and a random-intercept logistic regression model for binary data [6].

Results are presented as mean differences (MD) or odds ratios (OR) with 95% confidence intervals (CI). Analyses were conducted using the statistical programme R version 3.4.1 [19]. For study specific estimates please contact AFS.

## Results

Participant level data were available from up to 246,355 individuals, and were supplemented by summary effect estimates from data repositories, resulting in a sample size of 320,170 individuals, including 95,865 cases of MI, 16,437 stroke, 11,920 ischemic stroke, 51,623 T2DM, 54,702 cancer, 25,630 Alzheimer’s disease and 12,412 of COPD.

### Lipid and apolipoprotein associations

As reported previously [14], the four *PCSK9* SNPs were associated with lower LDL-C blood concentrations ranging from − 0.02 mmol/L (95% CI -0.03, − 0.02) per allele for rs11583680 to − 0.34 mmol/L (95% CI -0.36; − 0.32) for rs11591147 (See Additional file 2: Figure S1). *PCSK9* SNPs associated with a lower LDL-C concentration were also associated with lower concentrations of apolipoprotein B proportionate to the LDL-C association.

Associations of the GS with the other lipids or apolipoproteins, scaled to a 1 mmol/L lower LDL-C were (Table 1): 0.05 mmol/L (95% CI 0.02, 0.07) for HDL-C, − 0.07 mmol/L (95% CI -0.12, − 0.01) for TG, − 1.06 mmol/L (95% CI -1.12, − 1.00) for TC, − 0.20 g/L (95% CI -0.25, − 0.18) for ApoB, 0.02 g/L (95% CI -0.01, 0.06) for ApoA1, and − 4.12 mg/dL (95% CI -8.62, 0.38) for Lp(a).

**Table 1:** Biomarker associations of a *PCSK9* gene centric score, effect presented as mean difference (MD) with 95% confidence interval in brackets with the effects scaled to a 1 mmol/L decrease in LDL-C

Biomarker	Total sample size	MD (95% CI)
Lipids related biomarkers		
HDL-C in mmol/L	314,078	0.05 (0.02; 0.07)
TG in mmol/L	298,069	−0.07 (− 0.12; − 0.01)
TC in mmol/L	320,170	− 1.06 (− 1.12; − 1.00)
ApoA1 in g/L	55,477	0.02 (− 0.01; 0.06)
ApoB in g/L	54,643	−0.20 (− 0.25; − 0.18)
LP [a] in mg/dL	21,181	−4.12 (−8.62; 0.38)
Safety related biomarkers		
SBP in mmHG	182,487	0.03 (−0.05; 0.10)
DBP in mmHG	182,497	0.08 (0.001; 0.15)
CRP in log (mg/L)	91,990	0.03 (−0.07; 0.14)
IL-6 in log (pmol/L)	22,370	−0.08 (− 0.21; 0.04)
GGT in log (IU/L)	69,488	0.03 (−0.04; 0.10)
Fibrinogen in log(g/dL)	63,288	0.02 (−0.01; 0.04)
Hemoglobin in g/L	52,109	1.16 (−0.38; 2.70)
ALT in log (IU/L)	83,223	0.03 (−0.02; 0.08)
AST in log (IU/L)	49,556	0.01 (−0.03; 0.05)
ALP in log (IU/L)	60,222	−0.06 (− 0.09; − 0.02)
Creatinine in umol/L	100,206	0.06 (−1.43; 1.55)

Nota bene, *TG* triglycerides, *TC* Total cholesterol, *ApoA1* Apolipoprotein A1, *ApoB* Apolipoprotein B, *LPa* Lipoprotein a, *SBP* Systolic blood pressure, *DBP* Diastolic blood pressure, *CRP* C-reactive protein, *IL-6* Interleukin-6, *GGT* Gamma-glutamyltransferase, *ALT* Alanine transaminase, *AST* Aspartate transaminase, *ALP* Alkaline phosphatase

**Fig. 1:**
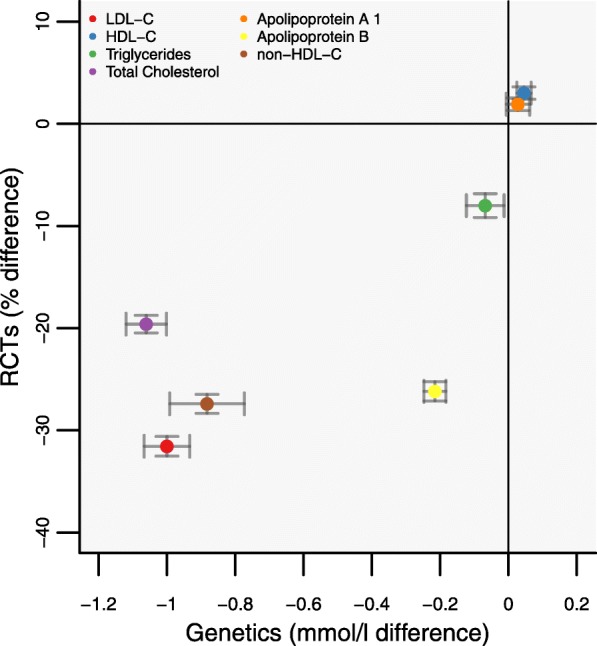
Lipid and lipoprotein associations of a *PCSK9* gene-centric score (GS) compared to placebo-controlled randomized trials of therapeutic inhibition of PCSK9. Footnote: Effect estimates are presented as mean differences, with 95% confidence interval (CI). Trial estimates are presented as percentage change from baseline (during 6 months of follow-up), and GS estimates scaled to a 1 mmol/L lower LDL-C (mmol/L). Results are pooled using a fixed effect model. Trial estimates are based on the systematic review by Schmidt et al 2017 [6, 17]

The associations of the *PCSK9* GS with blood-based lipid markers were directionally concordant with effects from treatment trials of therapeutic inhibition of PCSK9 (Fig. 1).

### Genetic associations with other biochemical and physiological measures

The GS estimates with SBP and DBP were 0.03 mmHg (95% CI -0.05, 0.10) and 0.08 mmHg (95% CI 0.0001, 0.15), respectively, per 1 mmol/L lower LDL-C. The *PCSK9* GS was associated with nominally lower ALP (IU/L) -0.06 (95% CI -0.09, − 0.02), but not with other liver enzymes (Table 1).

### Genetic associations with ischemic cardiovascular events

**Fig. 2:**
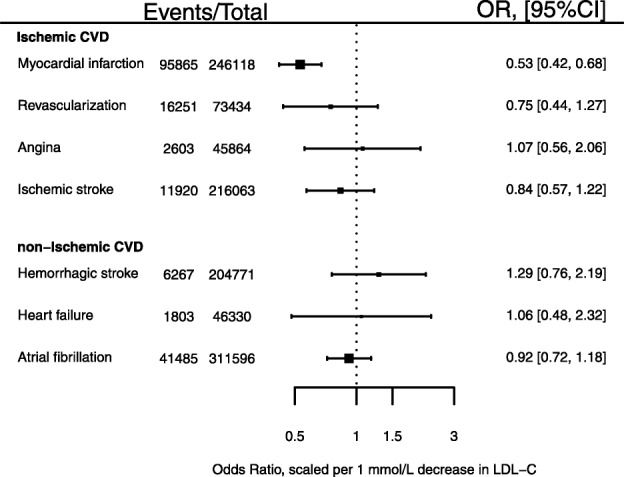
Associations of a *PCSK9* gene-centric score with ischemic and non-ischemic cardiovascular endpoints. Footnote: Effect estimates are presented as odds ratios (OR), with 95% confidence interval (CI) scaled to a 1 mmol/L lower LDL-C (mmol/L). Results are pooled using a fixed effect model. The size of the squares are proportional to the inverse of the variance

**Fig. 3:**
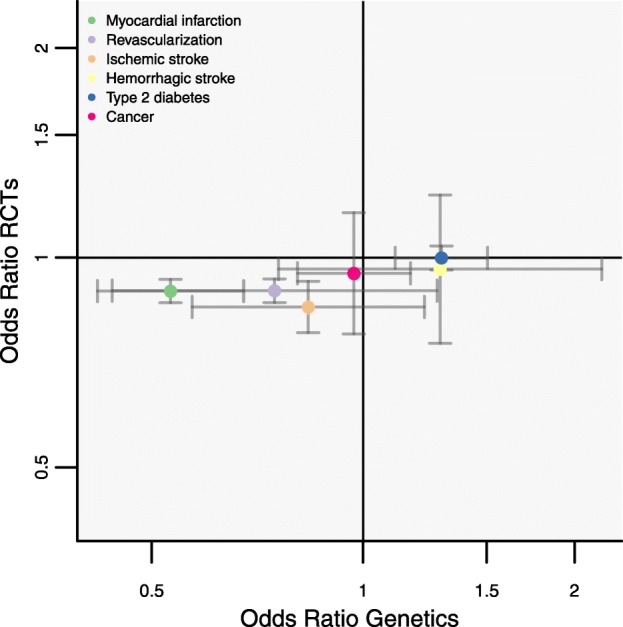
Clinical endpoint associations of the *PCSK9* gene-centric score (GS) as compared to placebo-controlled randomized trials of therapeutic inhibition of PCSK9. Footnote: Effect estimates are presented as odds ratios (OR), with 95% confidence interval (CI), for the GS scaled to a 1 mmol/L lower LDL-C (mmol/L). Results are pooled using a fixed effect model. Trial estimates are based on the systematic review by Schmidt et al 2017 [6], with the estimates on ischemic stroke and revascularization solely based on the FOURIER and ODYSSEY OUTCOMES trials

The *PCSK9* GS was associated with a lower risk of MI (OR 0.53; 95% CI 0.42; 0.68; 95,865 cases), which was directionally consistent with results from placebo-controlled PCSK9 inhibition trials: OR 0.90 (95% CI 0.86, 0.93), with both estimates scaled to a 1 mmol/L lower LDL-C (Figs. 2 and 3). The genetic effect estimate for ischemic stroke was OR 0.84 (95% CI 0.57, 1.22, 11,920 cases), concordant in direction to that of the drugs trials (OR 0.85 95% CI 0.78; 0.93). Similarly, the *PCSK9* GS association with coronary revascularization (OR 0.75 95% CI 0.44; 1.27) was directionally consistent with the PCSK9 inhibitor trials (OR 0.90; 95% CI 0.86, 0.93) (Fig. 3).

### Genetic associations with non-ischemic cardiovascular disease

The point estimate for the GS association with hemorrhagic stroke (Fig. 2), OR 1.29 (95% CI 0.76, 2.19), was discordant to the estimate from PCSK9 inhibitor trials (OR 0.96 95% CI 0.75; 1.23) (Fig. 3), although the confidence intervals overlapped. Comparing the association of *PCSK9* GS with hemorrhagic and ischemic stroke indicated the GS had a differential effect (*p*-value = 0.02). No *PCSK9* GS association was observed with atrial fibrillation (OR 0.92 95% CI 0.72; 1.18; 41,485 cases), or heart failure (OR 1.06 95% CI 0.48; 2.32; 1803 cases) (Fig. 2).

### Associations with non-cardiovascular disease and related biomarkers

**Fig. 4:**
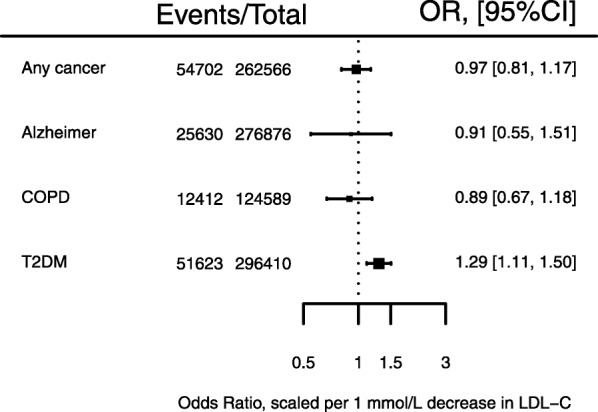
Associations of a *PCSK9* gene-centric score (GS) with non-cardiovascular events. Footnote: Effect estimates are presented as odds ratios (OR), with 95% confidence interval (CI) scaled to a 1 mmol/L lower LDL-C (mmol/L). Results are pooled using a fixed effect model. The size of the squares are proportional to the inverse of the variance. Note, that all GS estimates are based on 4 SNPs, except for the Alzheimer’s disease estimate which excluded the SNP rs11591147 due to lack of data

The *PCSK9* GS was not associated with the risk of any cancer (OR 0.97: 95%CI 0.81; 1.17; 54,702 cases, see Fig. 4), nor with any of 12 specific types of cancer (Additional file 2: Figure S2). We did not observe an association with either Alzheimer’s disease or cognitive performance: for Alzheimer’s the OR was 0.91 (95% CI 0.55, 1.51) and for cognition (per standard deviation) -0.03 (95% CI -0.22, 0.16). As reported before [14] the GS was associated with T2DM (OR 1.29 95% CI 1.11; 1.50) (Fig. 4), higher body weight (1.03 kg, 95% CI 0.24, 1.82), waist to hip ratio 0.006 (95% CI 0.003, 0.011) and fasting glucose 0.09 mmol/L (95% CI 0.02, 0.15). The OR for COPD was 0.89 (95% CI 0.67, 1.18).

## Discussion

The genetic findings presented here show that variation in *PCSK9* is associated with lower circulating LDL-C and apoB concentrations, lower risk of MI and, with lesser confidence, the risk of ischemic stroke and coronary revascularization. These effects are consistent in direction to effects observed in PCSK9 inhibitor trial’s [20].

A recent systematic review of trial data [21] indicated PCSK9 inhibition was associated with increased fasting glucose (0.17 as standardized mean difference [SMD] 95% CI 0.14; 0.19) and glycosylated haemoglobin (0.10 SMD 95% CI 0.07, 0.12, 21), although these associations were dependent on the inclusion of the terminated bococizumab trials. Recently we, and others, showed natural genetic variation *PCSK9* was associated with elevated fasting glucose and T2DM [14, 22, 23] and that variation at other LDL-C-associated loci also influence risk of T2DM [24, 25]. However, the FOURIER and ODYSSEY OUTCOMES trials, the largest treatment trials of PCSK9 inhibitors to date, did not find an association with risk of incident T2DM, at a median follow up of 2.2 and 2.8 years respectively. It is possible this reflects a genuine discordance between the findings from trials and genetic analyses. Alternatively, the exposure durations in the two largest trials may simply have been too short for subjects to develop T2DM. The risk increasing effect of statins on T2DM was only apparent after conducting a meta-analysis of 13 statin trials in which 4278 T2DM cases were observed during an average follow-up of 4 years [26].

In general, inconsistencies between associations of variants in a gene encoding a drug target and the effects of the corresponding treatment are possible on a number of theoretical grounds. The effects of genetic variation (present from conception) may be mitigated by developmental adaptation or environmental changes. A lack of association of a genetic variant with an outcome therefore does not preclude an effect of a treatment administered in later life, when adaptive responses may no longer be available, or in the presence of a particular environment [27]. We selected a subset of all genetic variants at *PCSK9* that capture information on many others and which have some annotated function. However, other approaches to more fully capture the entire gene-centric effect are worthy of future investigation [28].

The association of *PCSK9* variants with LDL-C and MI has been reported before [5], and was a motivating factor for the development of PCSK9 inhibiting drugs. Lotta and colleagues [22] reported a similar OR for MI of 0.60 (95% CI 0.48, 0.75) per 1 mmol/L decrease in LDL-C using the *PCSK9* rs11591147 SNP. Using a seven SNP *PCSK9* GS, Ference et al. reported a MI OR of 0.44 (95% CI 0.31, 0.64) per 1 mmol/L decrease in LDL-C [23]. These scaled genetic effects are larger than the treatment effect observed in trials which others have noted previously [29], and ascribed to the lifelong effect of genetic variation versus the short-term effect of drug treatment in later life.

The available trial data showed PCSK9 inhibitors had a similar effect on MI (OR 0.90, 95% CI 0.86; 0.93) and ischemic stroke (OR 0.85 95% CI 0.78; 0.93). By contrast, the genetic analysis indicated a directionally concordant, but larger effect on MI (OR 0.53; 95% CI 0.42; 0.68) than ischemic stroke, (OR 0.84 95% CI 0.57; 1.22). The genetic analysis was, however, based on only 11,920 stroke cases, about one-fifth of the number of cases available for the genetic analysis of MI and as such confidence interval overlapped. We did observe a differential association between *PCKS9* SNPs and ischemic and hemorrhagic stroke (interaction *p*-value = 0.02). Findings from statin trials previously suggested LDL-C lowering through inhibition of HMG-coA reductase is associated with a reduced risk of ischemic but potentially increased risk of hemorrhagic stroke [30–32]. Our findings suggest that a different effect on ischemic and hemorrhagic stroke subtypes may be eventually identified for PCSK9 inhibitors.

Despite previous concerns about a potential effect of this class of drugs on cognition [33], the genetic analysis did not reveal a significant association of the *PCSK9* variants with cognitive function or Alzheimer’s disease, nor with COPD or cancer, though this does not preclude an effect on such outcomes from drug treatment given in later life. While we explored the associations with any cancer (54,702 events) as well as individual cancer sites (Additional file 2: Figure S2), we did not have data on some clinically relevant cancer types such as endometrial cancer.

This neutral effect on cognition has been recently reported by the EBBINGHAUS study, nested within the FOURIER trial, which reported a non-significant PCSK9 inhibitor effect on multiple measures of cognition confirming (using a non-inferiority design) an absence of effect [33]; it should be noted that similar to the FOURIER, the EBBINGHAUS follow-up time was limited. The absence of an effect on cognition during PCSK9 inhibitor treatment was also observed in the ODYSSEY OUTCOMES trial, which had a median follow-up [7] of 2.8 years.

Drugs (even apparently specific monoclonal antibodies) can exert actions on more than one protein if such targets belong to a family of structurally similar proteins. PCSK9, for example, is one of nine related proprotein convertases [34]. Such ‘off-target’ actions, whether beneficial or deleterious, would not be shared by variants in the gene encoding the target of interest. In addition, monoclonal antibodies prevent interaction between circulating PCSK9 and LDL-receptor and should not, in theory, influence any intracellular action of the protein [35].

Genetic association studies of the type conducted here tend to examine the risk of a first clinical event, whereas clinical trials such as ODYSSEY OUTCOMES focus on patients with established disease, where mechanisms may be modified. Proteins influencing the risk of a first event may also influence the risk of subsequent events, as observed in the case of the target of statin drugs that are effective in both primary and secondary prevention [1]. For this and other reasons [36–38], examination of the effects of *PCSK9* variants on the risk of subsequent CHD events in patients with established coronary atherosclerosis is the subject of a separate analysis led by the GENIUS-CHD consortium [38].

## Conclusions

*PCSK9* SNPs associated with lower LDL-C predict a substantial reduction in the risk of MI and concordant associations with a reduction in risk of ischemic stroke, but with a modestly increased risk of T2DM. In this preliminary analysis we did not observe associations with other non-cardiovascular safety outcomes such as cancer, COPD, Alzheimer’s disease or atrial fibrillation.

## Additional files

Additional file 1: Supplemental tables. (XLSX 62 kb)

Additional file 2: Supplemental figures and study acknowledgments. (PDF 154 kb)

## Data Availability

The datasets used and/or analysed during the current study are available from the corresponding author on reasonable request.

## Abbreviations

ALP: Alkaline phosphatase
ALT: Alanine aminotransferase
ApoA1: Apolipoproteins A1
ApoB: Apolipoproteins B
AST: Aspartate transaminase
CADD: Combined annotation dependent depletion
CHD: Coronary heart disease
CI: Confidence interval
COPD: Chronic obstructive pulmonary disease
CRP: C-reactive protein
CVD: Cardiovascular disease
DBP: Diastolic blood pressure
GGT: Gamma-glutamyltransferase
GLGC: Global lipids genetics Consortium
GS: Gene-centric score
HbA1c: Glycated haemoglobin
IL-6: Interluekin-6
IPD: Individual participant-level data
LD: Linkage disequilibrium
LDL-C: Low density lipoprotein-cholesterol
LPa: Lipoprotein a
mAbs: Monoclonal antibodies
MAF: Minor allele frequency
MD: Mean difference
MI: Myocardial infarction
OR: Odds ratio
SBP: Systolic blood pressure
SMD: Standardized mean difference
T2DM: Type 2 diabetes mellitus
TC: Total cholesterol
TG: Triglycerides
