# Supplementary material for: Phenome-wide association analysis of LDL-cholesterol lowering genetic variants in PCSK9
Source: BMC Cardiovasc Disord. 2019 Oct 29;19:240. doi: 10.1186/s12872-019-1187-z (PMC6820948; doi:10.1186/s12872-019-1187-z)
Supplement: Supplementary file 2 — Supplemental figures and study acknowledgments. (PDF 154 kb) [file 12872_2019_1187_MOESM2_ESM.pdf]

Phenome-wide association analysis of LDL-cholesterol lowering genetic variants in *PCSK9*

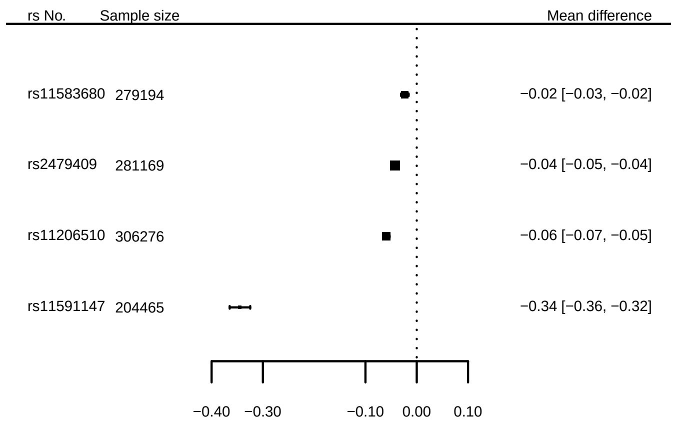

Appendix Figure 1: The LDL-C (mmol/L) effect of 4 *PCSK9* SNPs per LDL-C decreasing allele.

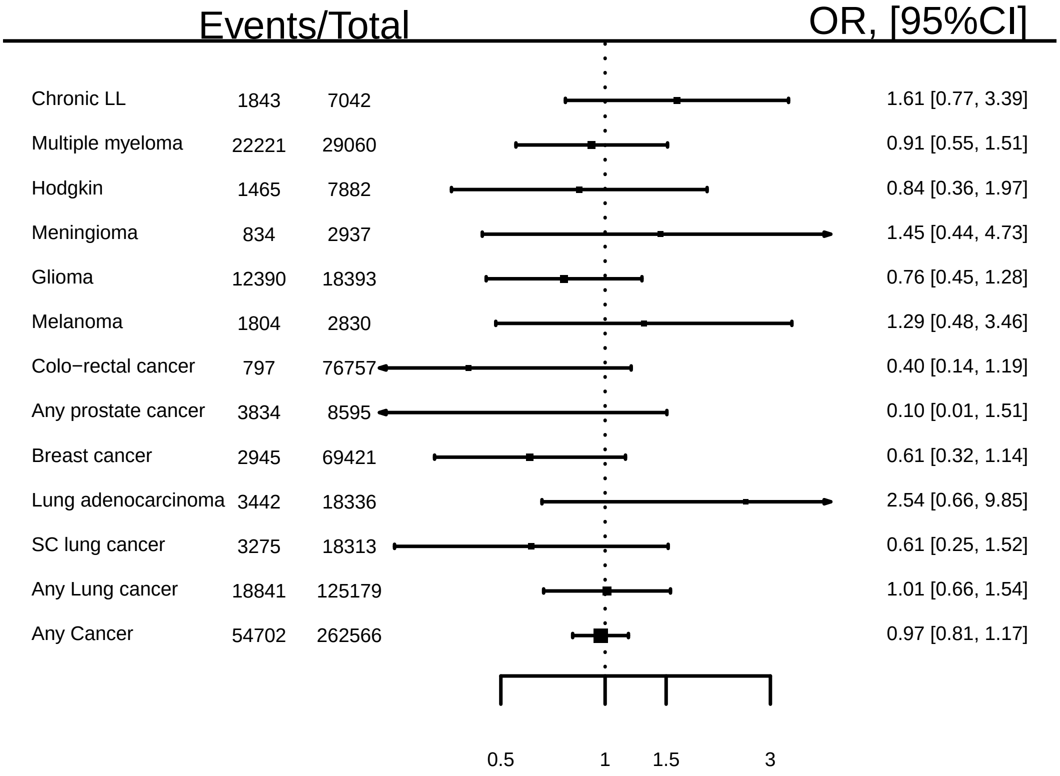

Appendix Figure 2: Associations of a PCSK9 gene-centric score (GS) with cancers. Effect estimates are presented as odds ratios (OR), with 95% confidence interval (CI) scaled to a mmol/L decrease in LDL-C (mmol/L). Results are pooled using a fixed effect model. The size of the black squares are proportional to the inverse of the variance.

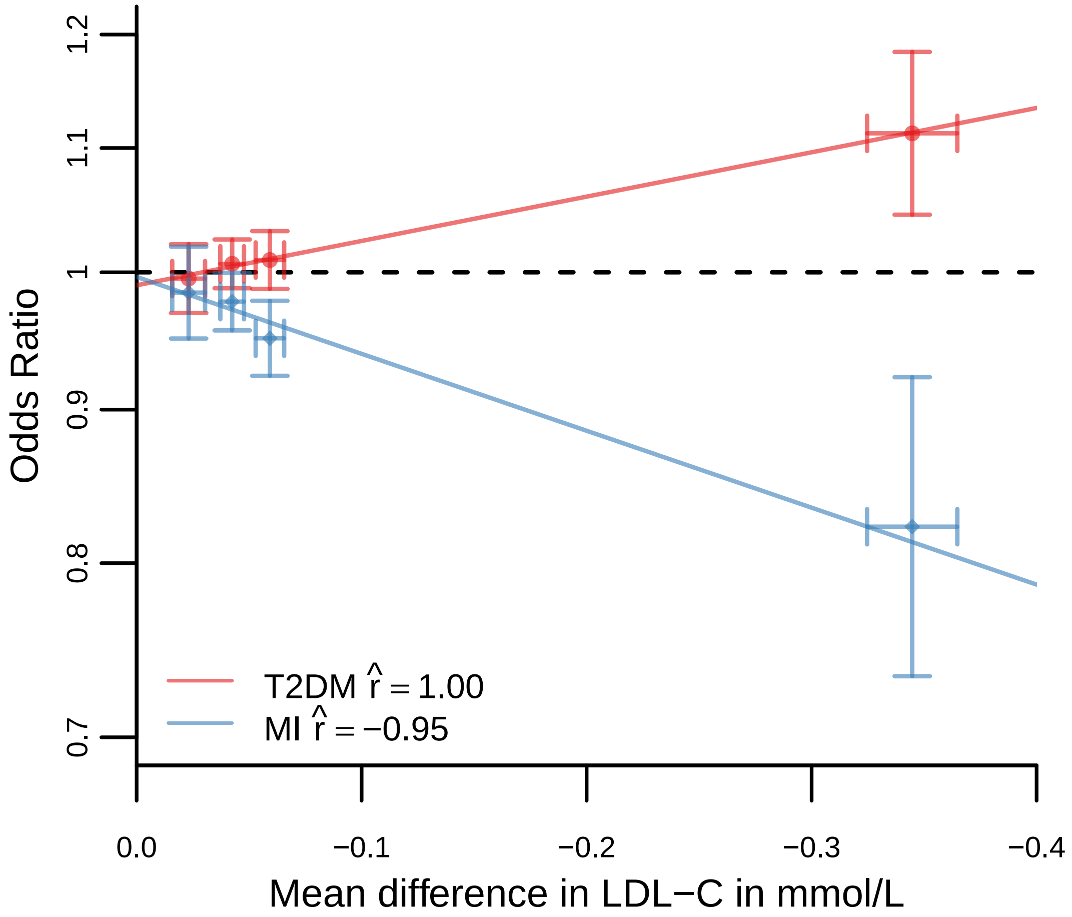

Appendix Figure 3: Associations of a PCSK9 gene-centric score (GS) with myocardial infarction or type 2 diabetes, and LDL-C. Effect estimates are presented as odds ratios (OR) or mean differences, with 95% confidence interval (CI). r = Pearson's correlation coefficient was estimated using a weighted linear regression.

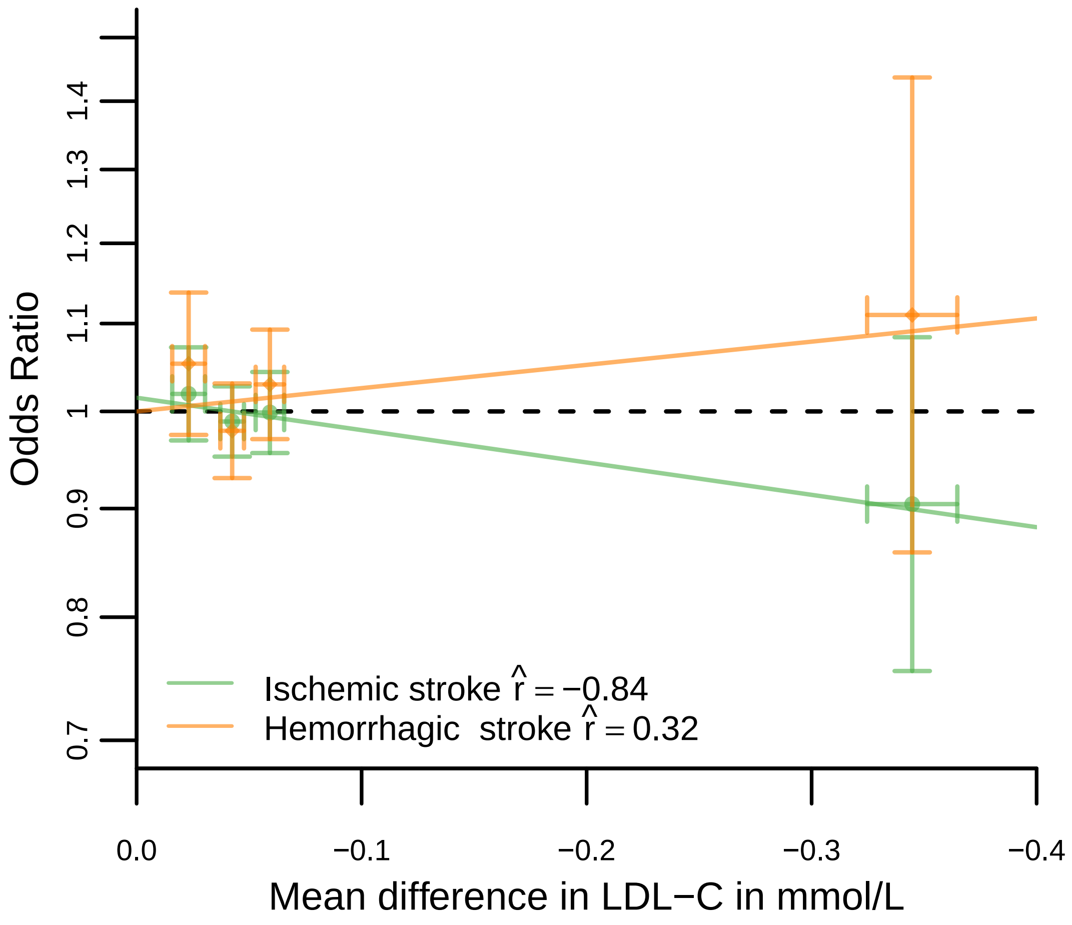

Appendix Figure 4: Associations of a PCSK9 gene-centric score (GS) with ischemic or hemorrhagic stroke, and LDL-C. Effect estimates are presented as odds ratios (OR) or mean differences, with 95% confidence interval (CI). r = Pearson's correlation coefficient was estimated using a weighted linear regression.

Individual study acknowledgments:

1958BC:This work made use of data and samples generated by the 1958 Birth Cohort (NCDS), which is managed by the Centre for Longitudinal Studies at the UCL Institute of Education. The authors are deeply grateful to the 1958 birth cohort participants for their long-standing commitment and support, and to all staff for cohort coordination and data collection. The management of the 1958 Birth Cohort is funded by the Economic and Social Research Council (grant number ES/M001660/1). Access to these resources was enabled via the 58READIE Project funded by Wellcome Trust and Medical Research Council (grant numbers WT095219MA and G1001799). DNA collection was funded by MRC grant G0000934 and cell –line creation by Wellcome Trust grant 068545/Z/02. This research used resources provided by the Type 1 Diabetes Genetics Consortium, a collaborative clinical study sponsored by the National Institute of Diabetes and Digestive and Kidney Diseases (NIDDK), National Institute of Allergy and Infectious diseases, National Human Genome Research Institute, National Institute of Child Health and Human Development, and Juvenile Diabetes Research Foundation International (JDRF) and supported by U01DK062418. This study makes use of data generated by the Wellcome Trust Case –Control Consortium. A full list of investigators who contributed to generation of the data is available from theWellcome Trust Case –Control Consortium website. Funding for the project was provided by the Wellcome Trust under award 076113. Support was provided by the National Institute for Health Research Biomedical Research Centre at Great Ormond Street Hospital for Children NHS Foundation Trust and University College London.

1982 Pelotas Birth Cohort: The 1982 Pelotas Birth Cohort Study is conducted by the Postgraduate Program in Epidemiology at Federal University of Pelotas (UFPel) in collaboration with the Brazilian Public Health Association (ABRASCO). From 2004 to 2013, the Wellcome Trust supported the 1982 Pelotas Birth Cohort Study. Additional funding was granted from The Brazilian National Research Council (CNPq) and Rio Grande do Sul State Research Support Foundation (FAPERGS). Previous phases of the study were supported by The International Development Research Center, World Health Organization, Overseas Development Administration, European Union, National Support Program for Centers of Excellence (PRONEX) and the Brazilian Ministry of Health. Genotyping was supported by the Department of Science and Technology (DECIT, Ministry of Health) and National Fund for Scientific and Technological Development (FNDCT, Ministry of Science and Technology), Funding of Studies and Projects (FINEP, Ministry of Science and Technology, Brazil), and Coordination of Improvement of Higher Education Personnel (CAPES, Ministry of Education, Brazil).

AMC–PAS: The AMC team is indebted to the participants of the AMC–PAS study.

BASE–II: BASE–II has been funded by the German Federal Ministry of Education and Research (BMBF) and has been formally divided into four subprojects: 'Psychology & Project Coordination and Database" (Max Planck Institute for Human Development [MPIB], grant number 16SV5837), 'Survey Methods and Social Science" (German Institute for Economic Research and Socioeconomic Panel [SOEP/DIW], grant number 16 SV5537), Medicine and Geriatrics (Charité—Universitätsmedizin, Berlin [Charité], grant number 16SV5536K), and 'Molecular Genetics" (Max Planck Institute for Molecular Genetics, now University of Lübeck [MPIMG–ULBC], grant number 16SV5538).

BiB study: Core support for Born in Bradford is provided by the Wellcome Trust (WT101597MA) and the National Institute for Health Research's Collaboration for Applied Health Research and Care (CLAHRC) for Yorkshire and Humber.

BRHS: We acknowledge the British Regional Heart Study team for data collection. The British Regional Heart study is supported by a British Heart Foundation grants (RG/08/013/25942)

and BHF (RG/13/16/30528). The British Heart Foundation had no role in the design and conduct of the study; collection, management, analysis, and interpretation of the data; preparation, review, or approval of the manuscript; and decision to submit the manuscript for publication.

Bruneck: K.W., S.K., and J.W. were supported by the Translational–Research–Programme grant ’Tyrol Score” funded by the ’Land Tirol” and an excellence initiative (Competence Centers for Excellent Technologies –COMET) of the Austrian Research Promotion Agency FFG: ’Research Center of Excellence in Vascular Ageing —Tyrol, VASCage” (K–Project Nr. 843536) funded by the BMVIT, BMWFW, the Wirtschaftsagentur Wien and the Standortagentur Tirol.

BWHHS: BWHHS is supported by funding from the British Heart Foundation and the Department of Health Policy Research Programme (England).

CAPS: CaPS was funded by the Medical Research Council and undertaken by the former MRC Epidemiology Unit (South Wales). The DNA bank was established with funding from a MRC project grant. The data archive is maintained by the University of Bristol.

CARe consortium including the ARIC, CARDIA, CHS, CFS, FHS, FHS offspring studies: The IBC array data (also known as Cardiochip or CVDSNP55v1_A from the National Heart, Lung and Blood Institute (NHLBI)Candidate Gene Association Resource (CARe) was downloaded with appropriate permissions from The database of Genotypes and Phenotypes (dbGaP) (www.ncbi.nlm.gov/gap).

Chronic lymphocytic leukaemia study: Funded by Bloodwise (formely Leukaemia Lymphoma Research) Fund (LRF05001, 06002 and 13044), Cancer Research UK (C1298/A8362), the Arbib Fund.

CoLaus: The CoLaus study was and is supported by research grants from GlaxoSmithKline, the Faculty of Biology and Medicine of Lausanne, and the Swiss National Science Foundation (grants 33CSCO–122661, 33CS30–139468 and 33CS30–148401).

Cyprus study: The Cyprus study was supported by a Cyprus Research Promotion Foundation grant (ERYEX/0406/06) and a joint Cyprus Research Promotion Foundation, Ministry of Health, and Cyprus Heart Foundation grant (41/5PE).

D.E.S.I.R. study: The D.E.S.I.R. study was supported by Inserm contracts with CNAMTS, Lilly, Novartis Pharma and Sanofi–Aventis, and by Inserm (Réseaux en Santé Publique, Interactions entre les déterminants de la santé, Cohortes Santé TGIR 2008), the Association Diabète Risque Vasculaire, the Fédération Française de Cardiologie, La Fondation de France, Société Francophone du Diabète, ONIVINS, Ardix Medical, Bayer Diagnostics, Becton Dickinson, Cardionics, Merck Santé, Novo Nordisk, Pierre Fabre, Roche and Topcon. The D.E.S.I.R. Study Group is composed of Inserm–U1018 (Paris: B. Balkau, P. Ducimetière, E. Eschwège), Inserm–U367 (Paris: F. Alhenc–Gelas), CHU d'Angers (A. Girault), Bichat Hospital (Paris: F. Fumeron, M. Marre, R. Roussel), CHU de Rennes (F. Bonnet), CNRS UMR–8199 (Lille: A. Bonnefond, P. Froguel), Medical Examination Services (Alençon, Angers, Blois, Caen, Chartres, Chateauroux, Cholet, LeMans, Orléans and Tours), Research Institute for General Medicine (J. Cogneau), the general practitioners of the region and the Cross–Regional Institute for Health (C. Born, E. Caces, M. Cailleau, N. Copin, J.G. Moreau, F. Rakotozafy, J. Tichet, S. Vol).

EAS: The Edinburgh Artery Study is funded by the British Heart Foundation (Programme Grant RG/98002), with Metabochip genotyping funded by a project grant from the Chief Scientist Office of Scotland (Project Grant CZB/4/672).

ELSA: Samples from the ELSA DNA Repository (EDNAR), received support under a grant (AG1764406S1) awarded by the National Institute on Ageing (NIA). ELSA was developed by a team of researchers based at the National Centre for Social Research, University College London and the Institute of Fiscal Studies. The data were collected by the National Centre for Social Research.

eMERGE: Electronic Medical Records and Genomics (eMERGE) Network was initiated and funded by the National Human Genome Research Institute (NHGRI), in conjunction with additional funding from the National Institute of General Medical Sciences (NIGMS) through the following grants: U01-HG-004610 (Group Health Cooperative/University of Washington); U01-HG-004608 (Marshfield Clinic Research Foundation and Vanderbilt University Medical Center); U01-HG-04599 (Mayo Clinic); U01HG004609 (Northwestern University); U01-HG-04603 (Vanderbilt University Medical Center, also serving as the Administrative Coordinating Center); U01HG004438 (CIDR) serving as Genotyping Center.

EPIC NL: The EPIC–NL study was funded by 'Europe against Cancer' Programme of the European Commission (SANCO), Dutch Ministry of Public Health, Welfare and Sports (VWS), Netherlands Cancer Registry (NKR), LK Research Funds, Dutch Prevention Funds, Dutch Cancer Society; ZonMW the Netherlands Organisation for Health Research and Development, World Cancer Research Fund (WCRF) (The Netherlands). Genotyping for the EPIC–NL study was funded by IOP Genomics grant IGE05012 from Netherlands Enterprise Agency (RVO).

EPICOR: The EPICOR study was supported by the Human Genetics Foundation (HuGeF) and Compagnia di San Paolo (to G.M.), Turin, Italy. EPIC–Italy is further supported by a grant from the 'Associazione Italiana per la Ricerca sul Cancro" (AIRC; to C.S.), and by Ministero dell'Istruzione, dell'Universitá e della Ricerca - MIUR project 'Dipartimenti di Eccellenza 2018 - 2022' to Department of Medical Sciences, University of Turin. The EPICOR authors wish to thank all the EPIC–Italy PIs and colleagues for their collaboration.

ET2DS: The Edinburgh Type 2 Diabetes Study is funded by the Medical Research Council (Project Grant G0500877); the Chief Scientist Office of Scotland (Programme Support Grant CZQ/1/38); Pfizer plc (Unrestricted Investigator Led Grant); and Diabetes UK (Clinical Research Fellowship 10/0003985). Research clinics were held at the Wellcome Trust Clinical Research Facility and Princess Alexandra Eye Pavilion in Edinburgh.

Fenland: The Fenland Study is funded by the Wellcome Trust and the Medical Research Council (MC_U106179471). We are grateful to all the volunteers for their time and help, and to the General Practitioners and practice staff for assistance with recruitment. We thank the Fenland Study Investigators, Fenland Study Co–ordination team and the Epidemiology Field, Data and Laboratory teams. We further acknowledge support from the Medical research council (MC_UU_12015/1).

Generation Scotland: Generation Scotland received core support from the Chief Scientist Office of the Scottish Government Health Directorates [CZD/16/6] and the Scottish Funding Council [HR03006]. Genotyping was funded by the Medical Research Council and the Wellcome Trust.

HAPIEE: The HAPIEE study was supported by Wellcome Trust 'Determinants of Cardiovascular Diseases in Eastern Europe: Longitudinal follow–up of a multi–centre cohort study" (The HAPIEE Project) (Reference number 081081/Z/06/Z); MacArthur Foundation 'Health and Social Upheaval (a research network)"; and National Institute on Aging 'Health disparities and

aging in societies in transition (the HAPIEE study)" (Grant number 1R01 AG23522).

Health 2006: The Health2006 was financially supported by grants from the Velux Foundation; The Danish Medical Research Council, Danish Agency for Science, Technology and Innovation; The Aase and Ejner Danielsens Foundation; ALK–Abello A/S, Hørsholm, Denmark, and Research Centre for Prevention and Health, the Capital Region of Denmark. This work was supported by the Timber Merchant Vilhelm Bang's Foundation, the Danish Heart Foundation (Grant number 07–10–R61–A1754–B838–22392F), and the Health Insurance Foundation (Helsefonden) (Grant number 2012B233). The Novo Nordisk Foundation Center for Basic Metabolic Research is an independent Research Center at the University of Copenhagen partially funded by an unrestricted donation from the Novo Nordisk Foundation (www.metabol.ku.dk).

HIFMECH: The HIFMECH study was supported by the European Commission (BMH4–CT96—0272), the Swedish Medical Research Council, the Swedish Heart—Lung Foundation, INSERM, Université de la Méditerranée (INSERM U626), Foundation pour la Recherche Médicale (FRM), and Programme Hospitalier de Recherche Clinique (PHRC 1996). HIFMECH co–investigators are A.H., S.E.H., Irène Juhan–Vague, Maurizio Margaglione, Giovanni di Minno, John Yudkin, and Elena Tremoli.

Hunter Community Study: The University of Newcastle provided $300 000 from its Strategic Initiatives Fund, and $600 000 from the Gladys M Brawn Senior Research Fellowship scheme; Vincent Fairfax Family Foundation, a private philanthropic trust, provided $195 000; The Hunter Medical Research Institute provided media support during the initial recruitment of participants; and Dr Anne Crotty, Prof. Rodney Scott and Associate Prof. Levi provided financial support towards freezing costs for the long–term storage of participant blood samples. The authors would like to thank the men and women participating in the HCS as well as all the staff, investigators and collaborators who have supported or been involved in the project to date.

Inter 99: The Inter99 was initiated by Torben Jørgensen (PI), Knut Borch–Johnsen (co–PI), Hans Ibsen and Troels F. Thomsen. The steering committee comprises the former two and Charlotta Pisinger. The study was financially supported by research grants from the Danish Research Council, the Danish Centre for Health Technology Assessment, Novo Nordisk Inc., Research Foundation of Copenhagen County, Ministry of Internal Affairs and Health, the Danish Heart Foundation, the Danish Pharmaceutical Association, the Augustinus Foundation, the Ib Henriksen Foundation, the Becket Foundation, and the Danish Diabetes Association.

INTERHEART and **INTERSTROKE**: Canadian Institutes of Health Research, Heart and Stroke Foundation of Canada, Canadian Stroke Network, Health Research Board Ireland, Swedish Research Council, Swedish Heart and Lung Foundation, The Health & Medical Care Committee of the Regional Executive Board, Region Västra Götaland (Sweden), AstraZeneca, Boehringer Ingelheim (Canada), Pfizer (Canada), MSD, Chest, Heart and Stroke Scotland, and The Stroke Association, with support from The UK Stroke Research Network.

JUPITER: The Justification for the Use of Statins in Prevention: an Intervention Trial Evaluating Rosuvastatin (JUPITER) was supported by AstraZeneca (to PMR). Genetic analysis in the trial population was supported by AstraZeneca (to DIC & PMR).

LifeLines: The LifeLines Cohort Study, and generation and management of GWAS genotype data for the LifeLines Cohort Study is supported by the Netherlands Organization of Scientific Research NWO (grant 175.010.2007.006), the Economic Structure Enhancing Fund (FES) of the Dutch government, the Ministry of Economic Affairs, the Ministry of Education, Culture and Science, the Ministry for Health, Welfare and Sports, the Northern Netherlands Collaboration of Provinces (SNN), the Province of Groningen, University Medical Center Groningen, the Uni-

versity of Groningen, Dutch Kidney Foundation and Dutch Diabetes Research Foundation. The authors wish to acknowledge the services of the Lifelines Cohort Study, the contributing research centers delivering data to Lifelines, and all the study participants.

NEO study: The Netherlands Epidemiology of Obesity (NEO) study: The NEO was designed for extensive phenotyping to investigate pathways that lead to obesity–related diseases. The NEO study is a population–based, prospective cohort study that includes 6,671 individuals aged 45—65 years, with an oversampling of individuals with overweight or obesity. At baseline, information on demography, lifestyle, and medical history have been collected by questionnaires. In addition, samples of 24–h urine, fasting and postprandial blood plasma and serum, and DNA were collected. Genotyping was performed using the Illumina HumanCoreExome chip, which was subsequently imputed to the 1000 genome reference panel. Participants underwent an extensive physical examination, including anthropometry, electrocardiography, spirometry, and measurement of the carotid artery intima–media thickness by ultrasonography. In random subsamples of participants, magnetic resonance imaging of abdominal fat, pulse wave velocity of the aorta, heart, and brain, magnetic resonance spectroscopy of the liver, indirect calorimetry, dual energy X–ray absorptiometry, or accelerometry measurements were performed. The collection of data started in September 2008 and completed at the end of September 2012. Participants are currently being followed for the incidence of obesity–related diseases and mortality.The authors of the NEO study thank all individuals who participated in the Netherlands Epidemiology in Obesity study, all participating general practitioners for inviting eligible participants and all research nurses for collection of the data. We thank the NEO study group, Pat van Beelen, Petra Noordijk and Ingeborg de Jonge for the coordination, lab and data management of the NEO study. The genotyping in the NEO study was supported by the Centre National de Génotypage (Paris, France), headed by Jean–Francois Deleuze. The NEO study is supported by the participating Departments, the Division and the Board of Directors of the Leiden University Medical Center, and by the Leiden University, Research Profile Area Vascular and Regenerative Medicine.

NPHS–II: The NPHS–II study was supported by the Medical Research Council, the US National Institutes of Health (NIH 33014), and Du Pont Pharma.

PIVUS: This project was supported by Knut and Alice Wallenberg Foundation (Wallenberg Academy Fellow), Swedish Diabetes Foundation (2013–024), Swedish Research Council (2012–1397 and 2015–02907), and Swedish Heart–Lung Foundation (20140422). Computations were performed on resources provided by SNIC through Uppsala Multidisciplinary Center for Advanced Computational Science (UPPMAX) under Project b2011036.

PREVEND: PREVEND genetics is supported by the Dutch Kidney Foundation (Grant E033), the National Institutes of Health (grant 2R01LM010098), The Netherlands organisation for health research and development (NWO–Groot grant 175.010.2007.006, NWO VENI grant 916.761.70, ZonMw grant 90.700.441).

SHIP and **SHIP–Trend** studies: SHIP is part of the Community Medicine Research net of the University of Greifswald, Germany, which is funded by the Federal Ministry of Education and Research (grants no. 01ZZ9603, 01ZZ0103, and 01ZZ0403), the Ministry of Cultural Affairs as well as the Social Ministry of the Federal State of Mecklenburg–West Pomerania, and the network ’Greifswald Approach to Individualized Medicine (GANI_MED)” funded by the Federal Ministry of Education and Research (grant 03IS2061A). Genome–wide data have been supported by the Federal Ministry of Education and Research (grant no. 03ZIK012) and a joint grant from Siemens Healthcare, Erlangen, Germany and the Federal State of Mecklenburg–West Pomerania. The University of Greifswald is a member of the Caché Campus program of the InterSystems GmbH.

TPT: We thank Professor Tom Meade for use of data from the Thrombosis Prevention Trial.

UCP: The UCP studies were funded by the Netherlands Heart Foundation and the Dutch Top Institute Pharma Mondriaan Project.

UDACS: Diabetes UK (BDA: RD01/0001357) supported the creation of UDACS.

UKHLS: UKHLS was funded by the Economic and Social Research Council (ES/M008592/1). The data were collected by NatCen and the genotyping was conducted by the Wellcome Trust Sanger Institute. Information on how to access the data can be found on the Understanding Society website https://www.understandingsociety.ac.uk/.

UHP: The Utrecht Health Project (UHP) is an ongoing dynamic population study initiated in a newly developed large residential area in Leidsche Rijn, part of the city of Utrecht and made possible by structural grants from the Julius Center.

WGHS: The Women's Genome Health Study (WGHS) was supported by grants from the National Heart, Lung, and Blood Institute (HL043851 and HL080467) and the National Cancer Institute (CA047988) to Julie E. Buring, with collaborative scientific support and funding for genotyping provided by Amgen (to PMR & DIC). The WGHS is currently supported by a grant from the NCI (UM1CA182913) to Julie E. Buring and I–Min Lee.

WHII: The WHII study is supported by grants from the Medical Research Council (G0902037; ID85374), British Heart Foundation (RG/07/008/23674), Stroke Association, National Heart Lung and Blood Institute (5RO1 HL036310), National Institute on Aging (5RO1AG13196) Agency for Health Care Policy Research (HS06516), and the John D. and Catherine T. MacArthur Foundation Research Networks on Successful Midlife Development and Socio–economic Status and Health.

WHI: The WHI program is funded by the National Heart, Lung, and Blood Institute, National Institutes of Health, U.S. Department of Health and Human Services through contracts HHSN268201100046C, HHSN268201100001C, HHSN268201100002C, HHSN268201100003C, HHSN268201100004C, and HHSN271201100004C.For a list of all the investigators who have contributed to WHI science, please visit: WHI inverstigators

Data on lipid traits have been contributed by the **GLGC** investigators and have been downloaded from http://csg.sph.umich.edu//abecasis/index.html. Data on glycemic traits have been contributed by the **MAGIC** investigators and have been downloaded from www.magicinvestigators.org. Data on anthropometric traits have been contributed by the **GIANT** investigators and have been downloaded from https://www.broadinstitute.org/collaboration/giant/index.php/Main_Page. Data on type 2 diabetes have been contributed by **DIAGRAM** investigators and Exome chip 80K downloaded from http://www.diagram–consortium.org/index.html and type 2 diabetes knowledge portal (http://www.type2diabetesgenetics.org/). We acknowledge the use of **SSGAC** cognition data http://www.thessgac.org/data. Data on coronary artery disease / myocardial infarction have been contributed by **CARDIoGRAMplusC4D** investigators and have been downloaded from www.CARDIOGRAMPLUSC4D.ORG.

LifeLines group authors

Behrooz Z Alizadeh (1), H Marike Boezen (1), Lude Franke (2), Pim van der Harst (3), Gerjan Navis (4), Marianne Rots (5), Harold Snieder (1), Morris Swertz (2), Bruce HR Wolffenbuttel (6), Cisca Wijmenga (2).

1. Department of Epidemiology, University of Groningen, University Medical Center Groningen, The Netherlands
2. Department of Genetics, University of Groningen, University Medical Center Groningen, The Netherlands
3. Department of Cardiology, University of Groningen, University Medical Center Groningen, The Netherlands
4. Department of Internal Medicine, Division of Nephrology, University of Groningen, University Medical Center Groningen, The Netherlands
5. Department of Medical Biology, University of Groningen, University Medical Center Groningen, The Netherlands
6. Department of Endocrinology, University of Groningen, University Medical Center Groningen, The Netherlands

UCLEB consortium authors

Borges C (1), Caddidy A (2), Charoen P (1), Chaturvedi N (3), Dale C (1), Drenos F (4), Dudbridge F (2), Engmann J (1), Finan C (1), Garfield V(5), Gaunt T (6), Gentry-Maharaj A (7), Jefferis B (8), Kuh D (9), Lawlor D (6), Mclachlan S (10), Menon U (7), Plagnol V (11), Price A (10), Sofat R (12), Talmud P (4), Tillin T (13), Walker A (4), White J (11), Whittaker J (14), Wong A (9).

1. Institute of Cardiovascular Science, University College London, UK
2. Dept Non-communicable Disease Epidemiology, London School of Hygiene and Tropical Medicine, UK
3. Cardiometabolic Phenotyping Group, Institute of Cardiovascular Science, University College London, UK
4. Centre for Cardiovascular Genetics, Dept. of Medicine, University College London, UK
5. Department of Epidemiology & Public Health, UCL Institute of Epidemiology & Health Care, University College London, UK
6. MRC Integrative Epidemiology Unit, School of Social and Community Medicine, University of Bristol, Bristol, UK
7. Institute for Women's Health, Faculty of Population Health Sciences, University College London, UK
8. Dept Primary Care & Population Health, University College London, UK
9. MRC Unit for Lifelong Health and Ageing, London, UK
10. Centre for Population Health Sciences, The Usher Institute of Population Health Sciences and Informatics, University of Edinburgh, UK
11. University College London Genetics Institute, Department of Genetics, Environment and Evolution, London, UK

12. Centre for Clinical Pharmacology, University College London, London, UK

13. Cardiometabolic Phenotyping Group, Institute of Cardiovascular Science, University College London, UK

14. Genetics Division, Research and Development, GlaxoSmithKline, Harlow, UK

The UCLEB consortium is supported by a British Heart Foundation Programme Grant (RG/10/12/28456).

AFGen Consortium

The AFGen Consortium was funded by the 1R01HL128914 & 2R01HL09257 grants.

METASTROKE acknowledgements

ASGC: Australian population control data were derived from the Hunter Community Study. We also thank the University of Newcastle for funding and the men and women of the Hunter region who participated in this study. This research was funded by grants from the Australian National and Medical Health Research Council (NHMRC Project Grant ID: 569257), the Australian National Heart Foundation (NHF Project Grant ID: G 04S 1623), the University of Newcastle, the Gladys M Brawn Fellowship scheme, and the Vincent Fairfax Family Foundation in Australia. Elizabeth G Holliday was supported by a Fellowship from the National Heart Foundation and National Stroke Foundation of Australia (ID: 100071).

BRAINS: Bio-Repository of DNA in Stroke (BRAINS) is partly funded by a Senior Fellowship from the Department of Health (UK) to P Sharma, the Henry Smith Charity and the UK-India Education Research Institutive (UKIERI) from the British Council.

GEOS: Genetics of Early Onset Stroke (GEOS) Study, Baltimore, USA was supported by the NIH Genes, Environment and Health Initiative (GEI) Grant U01 HG004436, as part of the GENEVA consortium under GEI, with additional support provided by the Mid-Atlantic Nutrition and Obesity Research Center (P30 DK072488), and the Office of Research and Development, Medical Research Service, and the Baltimore Geriatrics Research, Education, and Clinical Center of the Department of Veterans Affairs. Genotyping services were provided by the Johns Hopkins University Center for Inherited Disease Research (CIDR), which is fully funded through a federal contract from the NIH to the Johns Hopkins University (contract number HHSN268200782096C). Assistance with data cleaning was provided by the GENEVA Coordinating Center (U01 HG 004446; PI Bruce S Weir). Study recruitment and assembly of datasets were supported by a Cooperative Agreement with the Division of Adult and Community Health, Centers for Disease Control and Prevention and by grants from NINDS and the NIH Office of Research on Women's Health (R01 NS45012, U01 NS069208-01).

HPS: Heart Protection Study (HPS) (ISRCTN48489393) was supported by the UK Medical Research Council (MRC), British Heart Foundation, Merck and Co (manufacturers of simvastatin), and Roche Vitamins Ltd (manufacturers of vitamins). Genotyping was supported by a grant to Oxford University and CNG from Merck and Co. Jemma C Hopewell acknowledges support from the British Heart Foundation (FS/14/55/30806).

ISGS: Ischemic Stroke Genetics Study (ISGS)/Siblings With Ischemic Stroke Study (SWISS) was supported in part by the Intramural Research Program of the NIA, NIH project Z01 AG-000954-06. ISGS/SWISS used samples and clinical data from the NIH-NINDS Human Genetics

Resource Center DNA and Cell Line Repository (http://ccr.coriell.org/ninds), human subjects protocol numbers 2003-081 and 2004-147. ISGS/SWISS used stroke-free participants from the Baltimore Longitudinal Study of Aging (BLSA) as controls. The inclusion of BLSA samples was supported in part by the Intramural Research Program of the NIA, NIH project Z01 AG-000015-50, human subjects protocol number 2003-078. The ISGS study was funded by NIH-NINDS Grant R01 NS-42733 (J F Meschia). The SWISS study was funded by NIH-NINDS Grant R01 NS-39987 (J F Meschia). This study used the high-performance computational capabilities of the Biowulf Linux cluster at the NIH (http://biowulf.nih.gov).

MGH-GASROS: MGH Genes Affecting Stroke Risk and Outcome Study (MGH-GASROS) was supported by NINDS (U01 NS069208), the American Heart Association/Bugher Foundation Centers for Stroke Prevention Research 0775010N, the NIH and NHLBI's STAMPEED genomics research program (R01 HL087676), and a grant from the National Center for Research Resources. The Broad Institute Center for Genotyping and Analysis is supported by grant U54 RR020278 from the National Center for Research resources.

MILANO: Milano - Besta Stroke Register Collection and genotyping of the Milan cases within CEDIR were supported by the Italian Ministry of Health (Grant Numbers: RC 2007/LR6, RC 2008/LR6; RC 2009/LR8; RC 2010/LR8; GR-2011-02347041). FP6 LSHM-CT-2007-037273 for the PROCARDIS control samples.

WTCCC2: Wellcome Trust Case-Control Consortium 2 (WTCCC2) was principally funded by the Wellcome Trust, as part of the Wellcome Trust Case Control Consortium 2 project (085475/B/08/Z and 085475/Z/08/Z and WT084724MA). The Stroke Association provided additional support for collection of some of the St George's, London cases. The Oxford cases were collected as part of the Oxford Vascular Study which is funded by the MRC, Stroke Association, Dunhill Medical Trust, National Institute of Health Research (NIHR) and the NIHR Biomedical Research Centre, Oxford. The Edinburgh Stroke Study was supported by the Wellcome Trust (clinician scientist award to C Sudlow), and the Binks Trust. Sample processing occurred in the Genetics Core Laboratory of the Wellcome Trust Clinical Research Facility, Western General Hospital, Edinburgh. Much of the neuroimaging occurred in the Scottish Funding Council Brain Imaging Research Centre (www.sbirc.ed.ac.uk), Division of Clinical Neurosciences, University of Edinburgh, a core area of the Wellcome Trust Clinical Research Facility and part of the SINAPSE (Scottish Imaging Network-A Platform for Scientific Excellence) collaboration (www.sinapse.ac.uk), funded by the Scottish Funding Council and the Chief Scientist Office. Collection of the Munich cases and data analysis was supported by the Vascular Dementia Research Foundation. M Farrall and A Helgadottir acknowledge support from the BHF Centre of Research Excellence in Oxford and the Wellcome Trust core award (090532/Z/09/Z).

VISP: The GWAS component of the Vitamin Intervention for Stroke Prevention (VISP) study was supported by the United States National Human Genome Research Institute (NHGRI), Grant U01 HG005160 (PI Michèle Sale & Bradford Worrall), as part of the Genomics and Randomized Trials Network (GARNET). Genotyping services were provided by the Johns Hopkins University Center for Inherited Disease Research (CIDR), which is fully funded through a federal contract from the NIH to the Johns Hopkins University. Assistance with data cleaning was provided by the GARNET Coordinating Center (U01 HG005157; PI Bruce S Weir). Study recruitment and collection of datasets for the VISP clinical trial were supported by an investigator-initiated research grant (R01 NS34447; PI James Toole) from the United States Public Health Service, NINDS, Bethesda, Maryland. Control data obtained through the database of genotypes and phenotypes (dbGAP) maintained and supported by the United States National Center for Biotechnology Information, US National Library of Medicine.

WHI: Funding support for WHI-GARNET was provided through the NHGRI GARNET (Grant

Number U01 HG005152). Assistance with phenotype harmonisation and genotype cleaning, as well as with general study coordination, was provided by the GARNET Coordinating Center (U01 HG005157). Funding support for genotyping, which was performed at the Broad Institute of MIT and Harvard, was provided by the NIH Genes, Environment, and Health Initiative (GEI; U01 HG004424).

SiGN: The Stroke Genetics Network (SiGN) study was funded by a cooperative agreement grant from the National Institute of Neurological Disorders and Stroke (NINDS) U01 NS069208. Genotyping services were provided by the Johns Hopkins University Center for Inherited Disease Research (CIDR), which is fully funded through a federal contract from the National Institutes of Health (NIH) to the Johns Hopkins University (contract No.HHSN268200782096C). The Biostatistics Department Genetics Coordinating Center at the University of Washington (Seattle) provided more extensive quality control of the genotype data through a subcontract with CIDR. Additional support to the Administrative Core of SiGN was provided by the Dean's Office, University of Maryland School of Medicine. This work was supported by grants received from the German Federal Ministry of Education and Research (BMBF) in the context of the e:Med program (e:AtheroSysMed, the FP7 European Union project CVgenes@target (261123), the DFG as part of the CRC 1123 (B3), the Corona Foundation and the Fondation Leducq (Transatlantic Network of Excellence on the Pathogenesis of Small Vessel Disease of the Brain).

Online only methods

International Genomics of Alzheimer's Project (IGAP) is a large two-stage study based upon genome-wide association studies (GWAS) on individuals of European ancestry. In stage 1, IGAP used genotyped and imputed data on 7,055,881 single nucleotide polymorphisms (SNPs) to meta-analyse four previously-published GWAS datasets consisting of 17,008 Alzheimer's disease cases and 37,154 controls (The European Alzheimer's disease Initiative - EADI the Alzheimer Disease Genetics Consortium - ADGC The Cohorts for Heart and Aging Research in Genomic Epidemiology consortium - CHARGE The Genetic and Environmental Risk in AD consortium - GERAD). In stage 2, 11,632 SNPs were genotyped and tested for association in an independent set of 8,572 Alzheimer's disease cases and 11,312 controls. Finally, a meta-analysis was performed combining results from stages 1 & 2.
